# Supplementary material for: Are carbon emissions trading and green financial instruments synergistic? -Comprehensive quantitative research based on content analysis
Source: PLoS One. 2024 Mar 7;19(3):e0298601. doi: 10.1371/journal.pone.0298601 (PMC10919601; doi:10.1371/journal.pone.0298601)
Supplement: S1 Data — (ZIP) [file pone.0298601.s001.zip › Data/地级市Z_F工作报告.zip!/║■─╧/╒┼╝╥╜τ╩╨/└┤╫╘╣1⁄2╓┌║┼í╛┬φ┐╦╩2╛▌═°í┐_2011.docx]

各位代表：

现在，我代表市人民政府向大会作工作报告，请予审查，并请各政协委员和其他列席人士提出意见。

一、2010年及&ldquo;十一五&rdquo;时期工作回顾

2010年，是国内外发展环境极为复杂的一年，也是我市经济社会发展极不寻常的一年。全市上下在市委的统一领导下，认真贯彻中央和省委、省政府的决策部署，全面落实科学发展观，紧紧围绕建设世界旅游精品总目标，坚持以改革创新为动力，突出重点，踩准节奏，抢抓机遇，强基础、调结构、促转变、重民生、上水平，奋力推进&ldquo;五年大变样&rdquo;工程，全面实现了市五届人大二次会议确定的各项目标任务。

（一）经济增速创历史新高。2010年，预计（下同）全市实现生产总值241亿元，比上年增长15%，为建市以来最快增速，人均GDP达到2400美元；财政总收入达到20.82亿元，其中一般预算收入达到14.11亿元，分别增长20%；城镇居民人均可支配收入达到12700元,农民人均纯收入达到3600元,分别增长10.5%和10%；全社会固定资产投资增长24.6%，社会消费品零售总额增长19%，外贸进出口总额增长18.6%，分别达到143亿元、83.23亿元和2800万美元；居民消费价格涨幅控制在3%以内。在宏观政策偏紧的情况下，金融机构存、贷款余额分别增长20.9%和22.3%，位居全省前列；国土资源经营效益创历史新高，实现净收益5.56亿元；房地产业开发投资增长32.3%。

（二）产业转型升级取得实质性进展。游客接待规模和收入再创新高，分别达到2350万人次和125亿元，分别增长21.9%和24.8%；口岸扩大开放获得国务院批准，新开9个境外航点，入境游客达到140万人次，增长73%。中旅总社等10家全国百强旅行社和国际著名酒店品牌铂尔曼相继进驻，大成山水等高档酒店和黄龙洞生态广场等旅游休闲设施投入运营，旅游转型升级基础平台正逐步形成。成功协办首届中国国际文化旅游节，出色完成上海世博会展演任务，文化与旅游融合发展显示强劲活力，文化产业增加值占GDP的比重达到8.1%。旅游的繁荣带动整个服务业快速发展，第三产业增加值突破150亿元，同比增长15.1%。全市完成工业建设投资28.7亿元，同比增长30%，新增规模工业企业18家，实现全部工业增加值47亿元，同比增长18%；其中园区建设投资同比增长60.7%，新增入园企业17家。高新技术产业增加值增长25%。农业结构调整迈出新步伐，粮油面积减少、总产保持稳定，收购烤烟16.23万担，完成营造林14.7万亩，商品蔬菜面积达到40万亩，水果面积达到43万亩，农村承包土地依法流转面积达到16万亩，无公害农产品生产基地达到144.2万亩，各类农业休闲企业达到182家，67家省市级农业产业化龙头企业实现销售收入24亿元，全市农业增加值同比增长5.3%。

（三）城乡基础设施建设快速推进。面对诸多不利因素的制约，全市上下千方百计抓投资、上项目，建成张联公路、朱贺公路、市中心汽车站和桑植汽车西站，完成市博物馆主体工程。荷花机场、张花高速和张沅、慈石、桑鹤等干线公路建设不断加快，市城区贸易路、高云路等10条新建道路建成通车，北正街、南庄路等4条次干道和22条小街小巷改造完成，澧水风貌带、贺龙国际体育中心和武陵源原生态绿景园正式开工。城市环境综合治理&ldquo;穿衣戴帽&rdquo;工程取得新进展，户外广告规范整治取得明显成效。慈利澧水大桥和桑植帅乡路建成通车。建成农村通乡油路119公里、通村水泥路648公里，新增和改善农田灌溉面积2.18万亩,解决10.05万农村人口安全饮水问题。

（四）民本民生不断改善。32项省市为民办实事工程全面完成，新增城镇就业9600多人、农村转移就业4.6万人、各类社会保险参保4.06万人，建成保障性住房1854套，实现了城镇职工基本医疗市级统筹，退休职工养老金、在职职工最低工资、城乡最低生活保障、农村五保集中供养标准全面提高。义务教育合格学校和校舍安全工程年度建设任务已经完成，医药卫生体制改革逐步深化，乡镇文化站建设全面加强。市第四届全运会和第二届残运会成功举办。人口再生产保持低生育水平。生态环境质量进一步提高，建成慈利、桑植污水处理厂，万元GDP能耗和化学需氧量排放分别下降4.6%和5.3%，启动9个地质灾害应急防治项目建设，森林覆盖率和城市空气质量优良率分别达到69.6%和93.7%，武陵源区在全省率先建成国家级生态示范区。

（五）民主法制和精神文明建设全面加强。认真落实向人大及其常委会、人民政协和人大代表、政协委员报告、通报工作制度，积极实施重大问题集体决策和专家咨询、重大事项社会公示和听证制度，广泛邀请人大代表、政协委员、各界人士和市民代表列席政府常务会议，不断扩大人民群众的知情权，科学决策、依法行政、民主办事的水平不断提高。全面实施政府机构改革，依法依规科学制定和执行行政程序规定和规范行政裁量权办法，行政管理体制和运行机制不断创新完善。大力构建社会信用体系，社会诚信建设水平逐步提高。普法教育、社会治安综合治理和信访维稳工作力度加大，社会大局保持稳定。国防建设和军民共建取得佳绩，荣获全国&ldquo;人民防空先进单位&rdquo;和全省&ldquo;双拥模范城市&rdquo;称号。

一年来，全市上下团结拼搏，力图发展，各方面工作有效推进，监察、审计、统计、人事、编制、物价、国资监管、工商、税务、质量监督、食品药品卫生监督、商业保险、粮食、气象、外事侨务、贸促、民族、宗教、残联、移民、对台、档案、史志、无线电管理、新闻媒体等各项工作都取得了可喜的成绩。

各位代表，随着今年各项工作的收官，全市&ldquo;十一五&rdquo;经济社会发展的答卷圆满展现。盘点&ldquo;十一五&rdquo;，我们无比自豪和振奋。这五年， 虽然我们遭遇了特大冰雪冻雨灾害、汶川大地震、甲型H1N1流感、国际金融危机等连环冲击，经历了建市以来前所未有的多重考验，但在党中央、国务院和省委、省政府的正确领导下，我们团结和带领全市人民积极应对复杂多变的国内外环境，实现了前所未有的大发展。

这五年，是我市综合实力显著增强、经济发展最快的五年。生产总值净增133亿元，年均增长13.7%，比 &ldquo;十五&rdquo;时期年均快3.1个百分点。全市多项经济指标在&ldquo;十五&rdquo;期末的基础上翻了一番多：财政总收入和一般预算收入分别增长1.92倍，净增13.7亿元和9.3亿元；工业增加值增长1.15倍，净增28.15亿元；固定资产投资增长1.91倍，净增93.9亿元；社会消费品零售总额增长1.26倍，净增46.5亿元；居民储蓄存款增长1.85倍，净增108.4亿元；实际利用外资、省外内资分别增长2.21倍和2.8倍，进出口总额增长5.76倍，房地产开发投资增长5.08倍。尤其是文化产业与旅游融合发展，异军突起，一跃成为全省文化旅游发展的主力军和排头兵。五年间，我们相继打造了&ldquo;国际乡村音乐节&rdquo;、&ldquo;天门狐仙&middot;新刘海砍樵&rdquo;、&ldquo;张家界&middot;魅力湘西&rdquo;、&ldquo;武陵魂&middot;梯玛神歌&rdquo;等文化旅游优势品牌，获得了&ldquo;中国旅游文化示范地&rdquo;、&ldquo;文化强省先进集体&rdquo;等殊荣。

这五年，是我市基础设施建设取得重大突破、城乡面貌发生很大变化的五年。全市完成固定资产投资448亿元，是建市后前17年投资总和的1.5倍；争取建设用地指标2600公顷，年年实现耕地占补平衡。市城区市政基础设施建设实现投资43.7亿元，建成城市道路109公里、绿地274公顷，扩大建成区面积6.31平方公里，市工业园新区初现规模，全市城镇化率由32%上升到40.1%。五年来，我们完成荷花机场扩建一期工程和石怀铁路电气化改造，建成新火车站，新增等级公路1472公里，实现市治到区县一小时通勤，新建和改造农村公路5000公里，基本实现村村通公路、乡乡通油路目标；发电装机接近百万千瓦，城市供电可靠率达到99.9%，农村电网改造面和沼气普及率分别达到59%和26%；市城区和县城实现3G网络覆盖和有线电视数字化转换，建成电子政务外网平台，农村实现村村通电话、乡乡通宽带。全面完成111个村建设扶贫和第一轮新农村建设示范村创建任务，累计减少农村贫困人口8.97万人。

这五年，是我市民生建设扎实推进、群众得实惠最多的五年。全市累计投资36.72亿元，为人民群众办成了167项省定和17项市定实事。投入就业和社保资金30.14亿元，是&ldquo;十五&rdquo;时期的3.3倍，城乡就业和各类社会保险规模不断扩大，启动了新型农村养老保险试点，实现了城乡医保全覆盖、城乡低保应保尽保、乡乡镇镇有敬老院目标。投入社会发展资金49.6亿元，是&ldquo;十五&rdquo;时期的3.7倍，基本公共服务体系逐步建立和完善，人民群众上学、就医、娱乐、健身等生活条件明显改善。大力实施家电、汽车、摩托车、农机下乡和汽车、家电以旧换新工程，兑现财政补贴7377万元，直接拉动消费7亿多元。城乡居民收入年均增速比&ldquo;十五&rdquo;时期分别提高3个和5个百分点，人民群众的生活水平不断提升。

这五年，全市经济社会发展不断改观，而最深远的变化主要体现在三个方面：一是人民群众的文化观念正在发生着巨大变化，&ldquo;改革创新&rdquo;、&ldquo;开放包容&rdquo;意识不断增强，想变化、盼变化、干变化、支持变化已经成为全市上下的主流心声；二是我市经济社会整体架构和发展方式随着一大批国内外知名企业进驻落户，一大批重大基础项目相继建设和建成，正在逐步发生转变，民本民生、统筹协调发展也进一步得以加强；三是积极推进国际间文化交流、交往、交融，进一步扩大对外开放，创新营销城市，不断提升城市品牌品质，张家界对外的影响力、美誉度得以不断扩大和全面提升。

各位代表，这些成绩的取得来之不易，是党中央、国务院，省委、省政府正确领导的结果，凝聚了全市各族人民的辛勤劳动和聪明智慧，体现了全市上下勇克时艰、锐意进取的拼搏精神，展示了全市干部群众同心同德、奋发图强的崭新风貌。在此，我代表市人民政府，向全市各族人民，向驻张部队、武警官兵和政法干警，向各民主党派、工商联、人民团体和各界人士，向所有关心支持张家界发展的各位领导和国内外朋友，表示诚挚的感谢，并致以崇高的敬意！

各位代表，&ldquo;十一五&rdquo;的全面进步,标志着我们又站在了一个新的起点上。&ldquo;十一五&rdquo;的发展，不仅为我们新一轮又好又快的发展打下了坚实的物质技术基础，而且创造了丰富的精神财富，积累了弥足珍贵的经验。

第一，要坚持全面落实科学发展观，立足基本市情，抓住主要矛盾，扭住发展这个第一要务不松劲，坚定不移加快经济发展，坚定不移转变发展方式，以发展促转变，以转变促进更大发展。作为后发地区，我们的基本市情是基础差、底子薄，主要矛盾是发展速度不快、综合效益不高。加快发展和加快转变是我们推进世界旅游精品建设，打造国际风景旅游休闲度假城市的根本任务，是我们解决前进道路上各种困难和矛盾，促进社会和谐进步的关键条件，是我们实现富民强市，全面建设小康社会的基本途径，也是全市当前的最大政治。

第二,要坚持项目推动、投资拉动，积极运用世界眼光和战略思维，高起点、高标准、高质量谋划和推进重大项目建设，扩大有效投资规模，致力强基础、增实力，谋发展、促转变。这五年来，我市经济社会发展后劲逐步积累壮大，调结构、转方式的空间正在不断拓展，主要得益于这几年一大批事关全局和长远发展的重大基础设施、基础产业项目的有效实施，我市投资对经济增长的贡献充分显现，已达到60%以上。

第三，要坚持把解放思想、改革创新、扩大开放作为第一推动力，以解放思想统一发展意志、拓展发展眼界，以改革创新破解发展难题、强化发展保障，以扩大开放加快发展步伐、创造发展优势。这五年，我们不断创新发展理念和思路，绘就了城市发展、产业培育、新农村建设等一系列规划蓝图，发展的思路越来越清晰，发展的路子越来越宽阔；不断创新体制机制，实施了财政、交通、文化、城市建管、集体林权等多项体制改革，创新了旅游经营、招商引资、征地拆迁、社会管理、政务服务等多种制度规范，发展环境得到了有效改善；5家新的投融资平台开始给力，共实现我市建设融资70多亿元。

第四，要坚持人民政府关爱人民、服务人民、致富人民，切实践行&ldquo;发展为了人民、发展依靠人民、发展成果由人民共享&rdquo;的执政理念。只有把保障和改善民生作为政府一切工作的出发点和落脚点，我们才能真正代表人民群众的根本利益；只有充分尊重人民群众的首创精神，我们才能最广泛地凝聚发展的根本动力；只有不断满足人民群众对物质文化生活的新期待，我们才能有效激发全市上下共创美好明天的积极性、主动性和创造力。

第五，要坚持&ldquo;生态立市&rdquo;理念，致力打造绿色、低碳、宜居、宜游城市，加快建设&ldquo;两型社会&rdquo;，不断增创生态环保新优势。创造生态环保新优势，是转变经济发展方式的内在要求，是提升全市发展竞争力的现实途径。这五年，我们始终坚守生态环保这条生命线，大力推进生态建设，全面加强环境保护，投入财政资金4.67亿元，实现了节能减排目标，森林覆盖率和城市空气质量优良率分别提高4.3和3个百分点，环境质量水平保持全省领先地位，可持续发展能力进一步增强。

第六，要坚持科学决策、依法行政、民主办事，推崇求真务实、力戒空谈、取信于民，切实加强政府公信力和执行力建设。这是我们带领全市各族人民团结奋斗的重要保证。五年来，我们致力建设开放、务实、诚信、效率、廉洁的法治政府和服务型政府，以事业凝聚人心，用发展鼓舞人心，脚踏实地搞建设，一门心思干事业，治政水平和行政能力明显提高，干部群众团结拼搏的积极性汇成了推动全市经济社会发展的强大力量。

各位代表，回顾过去的五年，我们虽然取得了很大的成绩，但是，我们在前进的道路上仍然面临诸多矛盾、困难和问题。底子薄、基础差的基本市情还没有根本性改变，&ldquo;加快发展&rdquo;与&ldquo;加快转变&rdquo;矛盾十分突出。产业转型尚未取得根本性突破，经济发展的综合效益和水平不高；城镇化水平不高，交通等基础设施相对滞后，城市辐射带动能力不强，产业发展的物质技术平台仍显薄弱；城乡发展不平衡，农村处于贫困和温饱阶段的人口比重仍然较大；社会事业发展的历史欠帐较多，发展过程中各种冲突和博弈带来的社会矛盾日渐突出，在新时期如何正确看待人民内部矛盾，处理好经济发展与社会稳定的关系，把人民群众的积极性和创造力最大限度引导到加快发展上来，是对我们执政和治政能力的极大考验；还有一些旧的思想观念、思维方式和体制机制与科学跨越发展的要求不相适应，等等。这些问题，我们必须高度重视，认真对待，加以解决。

二、&ldquo;十二五&rdquo;时期的发展形势和任务

&ldquo;十二五&rdquo;时期，是我市加快建设世界旅游精品、实现富民强市的重要时期，也是我市强基础、增实力、调结构、转方式的关键时期。站在新的历史起点，纵观国际国内发展形势，我们既面临着新的发展机遇，又面临着新的困难挑战。当今世界正处于大发展大变革大调整之中，国际金融危机影响远没有消除，世界经济复苏动力不强、基础不牢、复杂多变。但是，世界多极化、经济全球化深入发展，和平、发展、合作仍然是时代潮流。我国综合实力和国际地位大幅提升，科学发展、加快转变经济发展方式成为推进发展的主题和主线，但经济社会正处于发展黄金期和矛盾凸显期交织阶段，转型中各种情况相互交错，面临诸多&ldquo;两难&rdquo;问题。就我市而言，作为后发地区的阶段性特征十分明显，基础条件薄弱，综合实力弱小、自我发展内生动力严重不足，但总体外部环境也为我们展现了很多机遇。&ldquo;十二五&rdquo;时期，中央将坚持实施扩大内需特别是消费需求战略，要加快旅游支柱产业培育发展；中央提出把实施西部大开发战略放在区域发展总体战略优先位置给予特殊政策支持，继续加大对革命老区、民族地区、贫困地区扶持力度，支持武陵山经济协作区和国家旅游综合改革试点城市建设；省委、省政府继续大力实施湘西地区开发，高度重视和大力支持我市加快世界旅游精品建设；特别是经过&ldquo;十一五&rdquo;大发展，全市各族人民对未来发展充满着新的企盼和期待。因此，&ldquo;十二五&rdquo;发展，我们既有压力更有动力，既有困难更有希望，既有挑战更有机遇，只要全市上下切实增强机遇意识、忧患意识和责任意识，努力提高驾驭经济社会发展大局和解决复杂问题的能力，善于在复杂的形势中抢抓和把握机遇，更加奋发有为地拼搏进取，就一定能够铸就张家界新的辉煌！

未来五年以及今后更长的时期，我市经济社会发展的中心任务，就是要把张家界建设成为&ldquo;功能齐全、交通便捷、产业发达、经济繁荣、人民富足、开放包容、文明和谐、具有优良的生态环境和特有的文化气质、绿色低碳、宜居宜游&rdquo;的世界旅游目的地。围绕这个中心任务，基于对国内外形势的分析判断和对全市经济社会发展实际的研究把握，提交本次大会审查的《张家界市国民经济和社会发展第十二个五年规划纲要（草案）》提出了今后五年发展的指导思想、奋斗目标、基本方略和战略重点。这是综合考虑各种因素作出的总体安排，具有较强的前瞻性、操作性和指导性。

（一）关于指导思想。纲要草案以求真务实的精神提出了全市经济社会&ldquo;十二五&rdquo;发展的指导思想，主要是要高举中国特色社会主义伟大旗帜，以邓小平理论和&ldquo;三个代表&rdquo;重要思想为指导，深入贯彻落实科学发展观，抓住一个主题，就是科学发展、富民强市；贯穿一条主线，就是加快经济发展方式转变；强化一个动力，就是改革、开放和创新；落实一个部署，就是要按照省委、省政府&ldquo;四化两型&rdquo;战略部署，坚持和完善&ldquo;1333&rdquo;发展思路，争取在全省率先建成&ldquo;两型社会&rdquo;；围绕一个目标，就是建设世界旅游精品这个总目标；坚持一条路径，就是&ldquo;以旅游为主导、新型工业强市、现代服务业兴市、加快城市建设、发展现代农业、促进扶贫攻坚&rdquo;的基本路径，调整经济结构，加快科技进步，推进节能环保，保障改善民生，两轮驱动，强基转型，统筹发展，强力推进旅游产业大市向旅游经济强市转变，努力实现富民强市新跨越。

贯彻这个指导思想，很重要的一条，就是要坚持&ldquo;两轮驱动&rdquo;。所谓&ldquo;两轮驱动&rdquo;，一是在一定时期内，我们还得依靠投资拉动，加快发展，依托本身产业基础引商引智，做大做强内生动力；二是要积极改革创新，推动发展方式转型转变。作为后发地区，二者相辅相成，缺一不可。加快发展是加快转变的基本前提。不加快发展，难以缩小差距并在新一轮激烈竞争中赢得主动权。而加快转变是科学发展观的内在要求，也是我们建设世界旅游精品的必然选择。不谋转变，难以全面提升经济发展质量和效益，做大做强综合经济实力；难以增创张家界发展新优势，实现可持续发展。在&ldquo;十二五&rdquo;时期，我们必须始终坚持在加快发展中强力推进发展方式的转变，以发展方式的转变促进我市更好更快的发展。

（二）关于奋斗目标。&ldquo;十二五&rdquo;时期全市经济社会发展的目标任务是：

&mdash;&mdash;综合实力明显增强。生产总值年均增长13%；人均生产总值突破4000美元，年均增长12%。财政总收入年均增长17%。实现全社会固定资产投资年均增长20%以上，力争&ldquo;十二五&rdquo;期间累计投资达到1400亿元；社会消费品零售总额年均增长18%；外贸进出口总额年均增长15%。旅游接待规模年均增长8%，过夜游客年均增长10%，入境游客年均增长15%，旅游收入年均增长15%；工业增加值年均增长20%；农业增加值年均增长5%。非公有制经济占GDP比重达到65%以上。力争到&ldquo;十二五&rdquo;期末，三次产业结构比调整为10:33:57。

&mdash;&mdash;社会事业更加繁荣。到2015年，力争城镇化率达到48%，中心城市人口达到30万人，建成区面积达到32平方公里；科技研发经费占GDP比重达到1.1%以上；学前三年教育毛入园率达到70%，九年义务教育完成率达到97%，高中阶段毛入学率达到86%，高等教育毛入学率达到35%；新型农村合作医疗覆盖率达到96%，城镇基本医疗保险覆盖率达到95%。

&mdash;&mdash;人口、资源和环境与经济发展日趋协调。到2015年，全市人口自然增长率控制在9&permil;以内，森林覆盖率达到71%，中心城市建成区绿化覆盖率达到50%，城市空气质量优良率达到98%，城市污水集中处理率和生活垃圾无害化处理率分别达到65%和80%。节能减排效果明显，资源节约和环境友好型社会初步建立。

&mdash;&mdash;城乡居民收入稳步增长。力争到2015年，城镇居民人均可支配收入、农民人均纯收入分别突破20000元和6000元，年均分别增长10%。居民消费价格涨幅控制在4%左右，城镇登记失业率控制在4%以内。

纲要草案提出的这个目标体系，是运用经济计量模型反复测算确定的，既保持了与&ldquo;十一五&rdquo;发展的承接延续，又考虑了发展的新形势新命题；既体现了加快发展的速度需要，又考虑了加快转变的质量要求；既突出了经济建设这个中心，又统筹了经济社会的协调发展。在此，我就几个主要指标问题作一下说明：

一是生产总值增长13%的问题。提出这个增速，一方面是基于对我市&ldquo;九五&rdquo;和&ldquo;十五&rdquo;年均增长10.6%、&ldquo;十一五&rdquo;年均增长13.7%的发展趋势的认识把握，另一方面是基于我市建设世界旅游精品和建设全面小康的现实要求，考虑全省及各市州的发展水平作出的分析判断。我们要想赶上或超过全省平均水平，缩小与发达地区差距，经济增长速度应该保持在13%以上。也只有保持这样的速度，我们才能凝聚人气、鼓舞人心，才能满足全市各族人民盼发展、求发展的新期待。

二是财政收入增长17%的问题。这既有现实的基础，也有实现的可能。&ldquo;十一五&rdquo;时期，我市财政总收入年均增长23.9%，&ldquo;十二五&rdquo;宏观经济形势和企业经营状况将趋于更好，重大项目建设力度将不断加大，价格总水平也会回升，有利于财政增收；同时按照财政收入适当高于经济增长才能支撑经济建设和社会发展的客观规律，我们迫切需要自加压力，做大财政收入总量。

三是投资力度问题。根据投资与GDP增长的弹性系数公式计算，我市近四年来投资弹性系数平均为1.85，即GDP每增长一个百分点，投资增长1.85个百分点。&ldquo;十二五&rdquo;时期，我们要实现生产总值年均增长13%，就必须有年均增长20%以上的投资予以支持。&ldquo;十二五&rdquo;期间，全市将建设一批事关经济社会发展大局的重大项目，着力构建与转方式、调结构、促发展相适应相配套的现代基础设施体系，投资增量将是非常明显的。

四是主导产业旅游发展问题。从我市旅游发展趋势来看，2010年我市旅游接待总规模达到创纪录的较高峰值，考虑到游客容量控制和&ldquo;十二五&rdquo;时期促进旅游转型升级的需要，我们不再追求过高的人次增长，更加注重客源结构的改善和综合效益的提高，重点开拓境外市场，促进高端消费。因此，纲要草案提出旅游接待总规模年均增长8%，入境游客年均增长15%以上。随着旅游产品体系不断完善，旅游与文化加快融合，游客逗留时间有潜力增加，预期过夜游客年均增长10%；旅游消费在转型升级进程中必将提高，预期旅游收入年均增长15%。经过努力，这些目标是可以达到的。

五是产业结构调整问题。纲要草案提出力争到&ldquo;十二五&rdquo;期末三次产业比调整为10:33：57。这样考虑，既是转方式、调结构所必需，也是实现富民强市目标之必然。特别是新型工业化，是经济发展不可逾越的阶段。经过近年来推进新型工业发展的积累，工业园区建设初见成效。&ldquo;十二五&rdquo;期间，我们必须采取超常规措施进一步加快新型工业发展，使之在调整结构、增加税收方面发挥更大的作用，促进整个产业结构优化升级。

六是城乡居民收入水平问题。纲要草案提出：&ldquo;十二五&rdquo;期间城乡居民收入年均分别增长10%，这主要是贯彻党的十七届五中全会&ldquo;实现居民收入增长和经济增长速度同步、劳动报酬增长和劳动生产率提高同步&rdquo;的要求，基于&ldquo;十一五&rdquo;我市城乡居民收入年均分别增长10.9%和10.1%的水平而拟订的。

七是生态环境指标问题。我们要实现13%的经济增速，就需要20%以上的投资增速，处理好经济建设与生态环境保护的关系至关重要。&ldquo;十一五&rdquo;时期，我市GDP增长与三类污染物排放量的弹性系数平均为0.25，由此推算，&ldquo;十二五&rdquo;我市三类污染物排放量每年将增长3.25%以上。因此，我们在推进经济社会发展的同时，必须强力推进环境保护和生态建设工程，确保实现经济社会发展和生态环境优化&ldquo;双赢&rdquo;目标。

(三)关于发展方略。确保&ldquo;十二五&rdquo;发展目标任务的顺利实现，我们必须坚持实施以下方略：

&mdash;&mdash;突出解放思想，正确处理经验传承和改革创新的关系。始终坚持把解放思想作为实现发展的首要任务，立足现实基础和成功经验，运用国际视野和战略眼光，不断推进改革创新，科学提升发展思路，加快构建促进更大更快更好发展的模式和体制机制，全面激发发展动力和活力。

&mdash;&mdash;突出旅游带动，正确处理旅游转型升级和总体经济结构调整的关系。旅游是我们的主导产业，旅游带动是我们的基础战略。调整产业结构必须首先做大做强、做优做特旅游产业，坚持以加快旅游国际化进程带动新型城镇化、新型工业化、农业现代化和信息化加快发展。

&mdash;&mdash;突出要素配置，正确处理强基转型与要素保障的关系。不断创新投融资方式和平台，多途径推进人才资源开发，科学统筹土地空间开发利用，全面增强能源供应能力，努力为强基转型提供资本、人才、土地、能源等要素支撑。

&mdash;&mdash;突出开放合作，正确处理内源性增长与外源性增长的关系。加强国际交流与合作，在国际间的交流、交融、交锋中提升城市发展境界、发展品质和国际化水平；加强区域交流与合作，在市场互通、资源互用、政策互动中抢占发展制高点；加强与国内外著名景区和企业交流与合作，大力引进新型战略合作者。

&mdash;&mdash;突出以人为本，正确处理推进发展与改善民生的关系。坚持把保障和改善民生作为一切工作的出发点和落脚点，着力完善比较完整、覆盖城乡、可持续的基本公共服务体系，着力建设绿色、低碳、宜居、宜游的美好家园，全面提高人民群众幸福指数。

（四）关于战略重点。纲要草案围绕加快推进世界旅游精品建设和富民强市，按照&ldquo;加快发展&rdquo;和&ldquo;加快转变&rdquo;两轮驱动的要求，紧扣发展的目标任务，提出了今后五年的五大战略重点：

1.着力调整经济结构，加快推进产业结构转型升级。产业结构不合理是我市经济发展最大的问题。我们必须把产业结构调整贯彻&ldquo;十二五&rdquo;发展始终，力争实现重大突破。一是以开展国家旅游综合改革试点为载体，加快发展休闲度假、商务会展、文化旅游等现代旅游业，大力推进旅游国际化进程，全面增强旅游产业素质和市场竞争力。二是按照&ldquo;资源与产品对接，项目向园区集中，产业成集群发展&rdquo;的要求，突出园区建设和引进、培育骨干企业，致力把新型工业打造成为加快发展的重要引擎，实现工业经济跨越发展。三是以市场化为导向，深化农业结构调整，实现&ldquo;四个农业&rdquo;专业化、标准化、规模化、集约化发展。四是把握国家扶持服务业大发展的黄金时期，充分发挥第三产业发展的相对优势，致力把现代服务业培育成为新的经济增长点。五是大力推进文化与旅游的深度融合，优化文化产业结构，力争把张家界打造成为湖湘文化的对外窗口，抢占全国文化旅游的制高点。

2.着力打造区域经济发展引擎，加快推进新型城镇化。新型城镇化是经济结构调整的战略重点，是破除城乡二元结构的根本途径，也是扩大内需、激发经济社会发展活力的重要引擎。&ldquo;十二五&rdquo;时期，我们规划的城镇化目标是48%，这就意味着有近一半的人口将工作和生活在城镇，必须加速推进以中心城市为核心、以县城为骨干、以建制镇和重点中心镇为支撑的新型城镇化进程。一是以打造国际风景旅游休闲度假城市为重点，全面实施新一轮城市规划和城乡一体化规划，加快推进中心城市和中心城镇基础设施和功能配套建设，大力打造旅游城市风貌特色；二是强力推动城乡区域协调发展，统筹城乡基础设施建设，统筹城乡产业发展，统筹城乡基本公共服务，推动城镇资本、技术、产业优势与农村资源优势相对接，形成以旅游业带动农业、以城市带动农村、城乡一体化发展的新格局。三是加快发展城市产业，大力培植新型工业，发展壮大城市休闲产业，加快发展商务会展产业，改造提升旅游美食产业，培育发展旅游城市创意产业，做大做强旅游房地产业，全面搞活城市经济。

3.着力增强经济社会发展后劲，加快推进重大基础设施建设。强基转型，是我们&ldquo;十二五&rdquo;时期的突出任务。全市上下要围绕强基转型，加快推进六大基础设施体系建设：一是加快构建现代交通体系。打造与重庆、武汉、贵阳等中心城市&ldquo;五小时交通经济圈&rdquo;，完善市域交通功能，基本形成四方通达、立体布局、方便快捷的现代交通网络。二是加快构建能源供应和资源配置体系。我市&ldquo;十一五&rdquo;时期GDP与能耗的弹性系数平均为0.66。&ldquo;十二五&rdquo;时期，全市GDP每年增长13%，则能耗每年需要增长8.58%，必须采取有效措施，确保能源供给平衡。&ldquo;十一五&rdquo;建设实践还表明，我市投资规模每增加一个百分点，新增城市建设用地需要增加0.41个百分点。以此测算，&ldquo;十二五&rdquo;期间，我市新增城市建设用地每年需增长5.35%以上，累计达到1846公顷左右；而目前省里给我市安排的用地指标只有950公顷，缺口将近一半。这个瓶颈，我们也必须千方百计予以突破。三是加快构建城乡水利保障体系。加强大中型水利基础设施建设，切实加强水资源保护，深入开展澧水流域综合治理开发，大力实施病险水库整治和城市防洪减灾、农田水利等配套工程建设。四是加快构建生态安全体系。大力推进生态保护和环境治理工程建设，大力发展绿色、低碳、循环经济，积极构建节约能源资源和保护生态环境的产业结构、增长方式和消费模式，全面打造资源节约型和环境友好型社会。五是加快构建&ldquo;数字城市&rdquo;管控体系，扎实推进社会信息化进程。六是加快构建社会公共安全防范体系，有效提高公共安全保障能力。

4.着力统筹城乡发展，加快推进新农村建设。农业农村问题始终是关系富民强市的全局性问题。没有农村的小康，就谈不上全面的小康；没有农民的富裕，就谈不上富民强市。&ldquo;十二五&rdquo;时期，我们必须大力调整农业结构，坚持以市场化为导向，以城市发展和旅游市场需要为引领，突出发展&ldquo;四个农业&rdquo;，加快传统农业向现代农业转变；必须围绕农业结构调整，加快农业农村基础设施建设，不断提高农业综合生产能力；必须深化农村综合改革，加快构建以农民合作经济组织为基础、骨干企业为龙头、公共服务机构为依托，公益性和经营性服务相结合的新型农业社会化服务体系；必须加大扶贫攻坚力度，坚持以桑植县为主战场，以产业开发为重点，努力探索扶贫开发与投入的新模式和新机制，取得更大更实的扶贫成效；必须着力推进新农村建设，精心组织实施4个省级新农村示范片建设，确保按期实现创建目标。

5.着力保障和改善民生，加快推进和谐社会建设。保障和改善民生，最重要的是加快推进基本公共服务均等化。一是加快推进社会事业与经济建设协调发展，大力实施建设教育强市战略，形成与全市跨越发展相适应的现代教育格局；大力实施科技强市战略，全面推进产学研结合，培育壮大科技创新能力；大力实施文化强市战略，深化文化体制改革，实现文化事业和文化产业大发展；大力实施医药卫生体制五项重点改革，全面提高人民群众医药卫生保障水平。二是始终把扩大就业放在提高人民生活水平的突出位置，强化政府促进就业的公共服务职能，不断完善劳动力市场体系，加大劳动力就业培训，多渠道增加就业岗位，实现经济增长与就业增长的良性互动。三是切实加强社会保障，全面提高保障水平。四是加强和创新社会管理，深入做好新形势下群众工作，深入推进民主法制和精神文明建设，深入开展&ldquo;平安满意在张家界&rdquo;专项活动，努力营造安定和谐的社会环境。

各位代表，&ldquo;十二五&rdquo;时期的发展蓝图是一个充满希冀的蓝图，也是一个具有挑战性的蓝图。实现这个蓝图，张家界必将迎来一个更加魅力无穷、幸福美好的明天！

三、2011年的预期目标和工作安排

各位代表，2011年是&ldquo;十二五&rdquo;发展规划实施的开局之年，又是我们全面实现&ldquo;五年大变样&rdquo;的收官之年，也是中国共产党成立90周年的大喜之年。明年中央将实行&ldquo;积极稳健，审慎灵活&rdquo;的宏观政策，我们在前进道路上将面临新的挑战与考验。全市上下要以党的十七届五中全会、省委九届十次全会和中央、省委经济工作会议精神为指导，深入贯彻落实科学发展观，正确把握国内外形势复杂变化的新特点，按照省委、省政府&ldquo;四化两型&rdquo;战略部署，在市委的统一领导下，紧扣建设世界旅游精品总目标，踩准节奏，找准路径，保增长，促转型，稳物价，惠民生，奋力完成&ldquo;五年大变样&rdquo;各项目标任务，为实施&ldquo;十二五&rdquo;规划起好步、开好局，以优异的成绩向党的90华诞献礼。

明年全市上下要努力实现以下主要预期目标：生产总值增长13.5%，其中一、二、三产业增加值分别增长5.5%、17%、14%；旅游接待人次和旅游收入分别增长8%和15%，过夜游客增长10%；全部工业和规模以上工业增加值增长20%；全社会固定资产投资增长20%以上；社会消费品零售总额增长18%；外贸进出口总额增长15%；财政总收入和一般预算收入分别增长17%和18%，城镇居民人均可支配收入和农民人均纯收入分别增长10%；居民消费价格涨幅控制在5%以内；新增城镇就业1万人，城镇登记失业率控制在4%以内，城镇化率达到41.7%；人口自然增长率控制在9&permil;以内，亿元GDP生产安全事故死亡人数控制在0.5人以内；节能减排实现省定目标，生态环境质量进一步提高；社会事业全面进步，民主法制和精神文明建设进一步加强，社会大局保持和谐稳定。

实现明年的目标任务，重点要抓好以下十个方面的工作：

（一）大力加快旅游产业转型提质。一是强力推进旅游转型基础项目建设。加快中心城市旅游特色街区、国际会展中心、高档度假酒店群、武陵源生态旅游度假区和高档体育文化娱乐产业项目建设，致力打造休闲度假、商务会展、文化演艺、康体养生、科考探险等现代旅游产品。精心打造文化产业，争创全国文化旅游优势品牌。二是培育壮大旅游市场主体。推动旅游龙头企业做大做强，扶持规范新兴旅游企业加快发展。三是继续创新旅游市场营销。重点抓好主题活动营销，办好国际乡村音乐节和全国皮划艇激流回旋锦标赛；充分利用覆盖面广、影响力大的媒体平台组织事件营销，不断制造具有轰动效应的兴奋点；继续推进&ldquo;靠大联强&rdquo;战略，力争在拓展欧美、台湾和东南亚等客源市场上取得重大突破。四是切实抓好旅游目的地建设和管理。全面启动国家旅游综合改革试点，科学编制精品旅游线路，统一营销旅游产品和规范旅游管理执法，建立健全旅游公共安全保障和应急服务体系，继续深化&ldquo;平安满意在张家界&rdquo;专项活动，不断提高旅游市场的美誉度和满意度。加强与世界旅游组织等涉旅国际机构合作，积极发展国外友好城市和景区关系，着力提升旅游目的地国际化水平。

（二）大力加强城镇建设和管理。按照新一轮城市总体规划，科学完善城市建设专业规划和控制性详规，实现城市控制性详规和规划法定图则管理全覆盖。继续调整和完善以县城关镇为重点的建制镇发展规划，力争城乡体系规划覆盖城乡和景区景点。围绕打造国际风景旅游休闲度假城市目标，加快澧水风貌带、贺龙国际体育中心等标志性工程建设，基本完成城市环境综合整治提升&ldquo;穿衣戴帽&rdquo;工程；实现鹭鸶湾大桥和西溪坪&ldquo;四路&rdquo;等15条城市道路建成通车，完成武陵源区武陵路、天子路扩建及改造任务，力争张清公路城市段改造工程开工；继续完善供水、排污、供电、通信和电子监控管线网络，扎实推进小街小巷整治和&ldquo;城中村&rdquo;改造。着力创新城市精细化管理模式，落实&ldquo;门前三包&rdquo;管理，推进城市标准化建设，提高城市管理品位。支持慈利、桑植新城区建设，开工修建永安和双门岛大桥，扶持江垭、瑞塔铺、沅古坪等重点建制镇发展，积极引导符合落户条件的农业人口向城镇转移。

（三）大力实施以重点工程为龙头的基础设施建设。一是全面加快&ldquo;五年大变样&rdquo;项目建设，确保按期优质完成任务。二是加快推进张花高速、荷花机场扩建二期、环武陵源景区公路和慈茶、桑鹤公路建设，实现慈石公路、张沅公路南段建成通车，启动永定城区绕城公路、旅游航道建设，力争黔张常铁路开工兴建。三是加快实施淋溪河电站、城乡电网、通信网络建设和改造工程，支持国家电网锦屏至苏南特高压直流输电线建设。四是扎实开展安张衡铁路、龙张高速、张桂高速、慈南高速、市城区至武陵源轻轨等项目和桑植新能源产业开发前期工作。五是坚持重大工程项目市级领导、市直部门联系责任制和定期调度制度，全面提升政务环境和施工环境质量。

（四）大力推进工业园区建设，促进新型工业快速成长。坚持把工业作为强基础、转方式、调结构的重要着力点，以更开放的思路、更有效的措施推进新型工业化。一要强力推进工业园区建设，力争新增入园企业20家，园区工业增加值增长40%。市工业园要在基础设施建设、项目引进落地、体制机制创新上实现大突破，慈利工业园要在改善整体形象、打造产业集群上取得大成效，桑植工业园要完成规划、进入实质性建设。二是大力培育优势产业和骨干企业，重点支持绿色清洁能源、生物有效成份提取、优势矿产精深加工和旅游特色商品产业发展，启动张家界软件园等龙头项目建设，力促奥威科技、远大住工、恒亮、港越等骨干企业加快发展。三是着力培育工业文化氛围。全面落实鼓励支持非公有经济发展的各项政策，推行现代企业制度，培育企业家队伍，努力营造&ldquo;开放、创新、超越&rdquo;的工业文化氛围。

（五）大力扩大现代农业规模，促进&ldquo;三农&rdquo;全面发展。稳定粮油生产，调整产业结构，提高农民收入，加大扶贫攻坚力度，加快发展县域经济，努力推进农业农村经济又好又快发展。一是依靠农业科技和农业机械化，提高粮油生产效益。二是加快推进&ldquo;四个农业&rdquo;集中连片发展，着力提高无公害蔬菜、优质干水果、优质烤烟、有机茶叶、花卉苗木、草食牲畜、特种养殖、休闲农业的规模效益。三是创新农业发展机制，建立健全农业服务体系。大力促进农民专业合作组织建设，有效扶持农业龙头企业做大做强。加强农产品质量监管，确保农产品质量安全。积极建立村镇银行，加强农业农村金融服务。加强农产品市场体系建设，切实搞活农产品流通。四是紧紧围绕农业结构调整布局，科学规划，统筹推进农村交通、农田水利、土地整理、农村能源、农电改造等基础设施建设。新建和改造乡村公路500公里、灌溉渠道380公里、沼气池5000口，新增农村安全饮水人口10万人。启动84个整村推进扶贫村和4个省级新农村示范片建设。

（六）大力发展现代物流，促进现代服务业做大做强。大力引进国内外知名品牌，支持运通物流园、华天商贸城、汽配城和4S店建设，建成维港十字街、溪布街和逸臣盛世旅游购物中心。加快工业园物流配套步伐，完成西溪坪仓储物流配送中心和慈利&ldquo;湘西东市&rdquo;一期工程建设。推进口岸全面开放，挂牌成立海关，增开货检业务，促进临空经济成长。加快&ldquo;数字城市&rdquo;建设，强化信息服务支撑。启动宽带、有线电视、电话&ldquo;三网融合&rdquo;工程，全面推进电子政务，积极发展电子商贸物流等新兴业态。做优做强旅游房地产业，全面提升社区服务业，积极开发养老服务业，充分发挥商业保险在经济社会发展中的重要作用。

（七）大力改善和保障民本民生。完善城乡公共就业服务体系，多渠道开发就业岗位。继续完善社会保障体系，编织更厚更实的社保安全网。明年，市人民政府在全面落实省定实事项目的基础上，继续集中财力为人民群众办好五件实事：一是继续资助200名家庭经济困难大学新生入学，二是新建100个治安电子防控系统，三是改造市城区30条小街小巷，四是综合整治城市污水管网10公里，五是解决好18个乡镇垃圾治理问题。保障和改善民本民生是政府工作永恒的宗旨。要大力实施建设教育强市战略，加强和规范学前教育，巩固和提升义务教育，提高高中教育质量，支持高校、职校、特校建设。建成市技工学校新校区，启动张家界航院新校区建设。大力落实科技&ldquo;市厅会商&rdquo;制度，实施知识产权战略，推进市场主体产学研相结合，提高科技创新能力。大力深化医药卫生和文化体制改革，繁荣体育事业，做好人口计生、民政民族、宗教事务、人民防空、国防教育以及妇女、儿童、老龄、残联等各项工作。要积极创新社会管理方式，强化社区服务和管理功能。建立社会稳定风险评估机制，严格国土资源、建设规划、安全生产、市场价格监管，抓好食品、药品、农产品放心工程，加强地震抗震设防和地质灾害防治工作。落实信访维稳分级负责制，加强流动人口管理、特殊人群帮教和社会舆论引导，有效化解各种社会不稳定因素。加强社会治安综合治理，及时有效打击各种违法犯罪。深入推进法制教育和精神文明建设，不断提高市民文明素质。

（八）大力创新推进科学跨越发展的体制机制。以推进国家旅游综合改革试点为契机，加快行政管理、经济体制和市场机制改革创新，继续推进国企改革和农村综合改革，全面完成集体林权制度改革。依照相关法律法规，积极探索健全征地拆迁安置补偿机制，有效培育被征地拆迁群众新的生产力。深入创建金融生态安全区，加快中小企业信用担保网络体系和小额贷款公司建设，促进各类融资平台良性发展。积极运用现代融资方式破解资金瓶颈，支持符合条件的企业通过股份制改造、发行债券和上市等途径直接融资，鼓励采取BOT、TOT等方式建设城镇基础设施和发展公用事业。不断创新对外开放理念，创新招商引资机制，积极承接国际国内资本和产业转移，力争在引进战略投资者上取得更大突破。

（九）大力加快生态市建设步伐。继续实施城乡绿化美化工程，以城市、景区、公路周边为重点，统筹林业生态建设。新增人工造林4.8万亩，完成古人堤绿化任务，力争建成回龙观公园，加强森林防火和松材线虫病防治。加大环境治理力度，严格保护世界自然遗产和地质遗迹，力争启动武陵源景区移民搬迁工程；继续推进城镇污水集中处理和垃圾无害化处理设施建设，强化重金属污染、涉及危险化学品企业和洗涤行业专项整治，积极实施农村垃圾治理&ldquo;三年行动计划&rdquo;，强化城郊、农村面源污染控制，加大矿山环境整治力度，完成省定节能减排任务。倡导低碳文明消费理念，推广使用清洁能源，促进再生资源回收利用。

（十）大力建设法治政府和服务型政府。一是加强民主法制建设，坚持依法行政。深入开展&ldquo;法治张家界&rdquo;创建活动，严格执行《湖南省行政程序规定》和《湖南省规范行政裁量权办法》，主动接受人大及其常委会、人民政协、民主党派和新闻媒体、社会公众的监督，让权力在阳光下运行。二是严格绩效评估考核，提高政府工作效率。加强机关效能建设，切实改进工作作风，落实评估结果运用，努力形成肯干事的人有机会、能干事的人有舞台、干成事的人有奔头的良好局面。三是坚持以人为本，认真开展新形势下群众工作。把握现代社会群众工作规律，自觉从群众利益出发想问题、作决策、做工作，充分体现人民政府同人民群众利益的一致性；完善社会管理网络，提高信访维稳工作水平，正确处理好人民内部矛盾。四是坚持从严治政，提升人民政府良好形象。积极开展学习型机关建设，全面提高公职人员队伍素质。充分发挥监察、审计等职能部门的作用，加强对行政权力运行的约束，真正树立起人民政府勤奋务实、清正廉洁的形象。

各位代表：历史的经验告诉我们，每一轮世界性的经济大调整，都将给一些地方脱颖而出、迎头赶上提供难得的契机。让我们在省委、省政府和市委的统一领导下，以只争朝夕的精神抢抓各种机遇，锐意开拓进取，为全面实现&ldquo;五年大变样&rdquo;目标、加快推进世界旅游精品建设而努力奋斗！
